# Body Mass Index Changes at 1.5 and 3 Years of Age Affect Adult Body Composition

**DOI:** 10.3390/pediatric16030056

**Published:** 2024-07-31

**Authors:** Chiharu Miyayama, Hiromichi Shoji, Yayoi Murano, Kanami Ito, Mizue Saita, Toshio Naito, Hiroshi Fukuda, Toshiaki Shimizu

**Affiliations:** 1 Department of Pediatrics, Juntendo University Faculty of Medicine, Tokyo 113-8421, Japan; ckojima@juntendo.ac.jp (C.M.); tshimizu@juntendo.ac.jp (T.S.); 2 Safety and Health Management Office, Hongo-Ochanomizu Campus, Juntendo University, Tokyo 113-8421, Japan; 3 Department of General Medicine, Juntendo University Faculty of Medicine, Tokyo 113-8421, Japan; 4 Department of Advanced Preventive Medicine and Health Literacy, Juntendo University Graduate School of Medicine, Tokyo 113-8421, Japan

**Keywords:** body mass index, Maternal and Child Health Handbook, adiposity rebound

## Abstract

Early childhood lays the foundation for many outcomes in later life. Recent studies suggest that early childhood development may contribute to lifestyle-related diseases such as obesity, type 2 diabetes, and cardiovascular disease in adulthood; however, there have been few investigations on this association among adults in Japan. Therefore, we examined the relationship between recent physical measurements in adults who underwent health checkups at our university and their physical measurements at birth and during infancy recorded in the Maternal and Child Health Handbook. The median age and body mass index (BMI) of the participants were 36 years and 20.4 kg/m^2^, respectively. BMI at the time of health checkup in adults did not correlate with physical measurements at birth, but it was found to be associated with BMI at 1.5 (regression coefficient (β) 0.53, *p* < 0.05) and 3 (β 0.7, *p* < 0.01) years of age. In addition, the waist-to-height ratio in adulthood was also associated with BMI at 1.5 (β 0.01, *p* < 0.05) and 3 (β 0.01, *p* < 0.05) years of age. These findings suggest that it is critical to provide appropriate guidance to children with high BMI and their parents during health checkups to prevent lifestyle-related disorders in adulthood.

## 1. Introduction

Since the 1990s, the concept of the Developmental Origins of Health and Disease (DOHaD) has attracted attention. The DOHaD suggest that exposure to challenging environments during critical periods of early life—specifically “the first 1000 days” from conception to 2 years of age—can predispose individuals to non-communicable diseases such as obesity, type 2 diabetes, and cardiovascular disease later in life. Prior to that, attention was focused mainly on the relationship between fetal nutritional status and disease, with Barker et al. [1] reporting in 1986 the outcomes of a large-scale epidemiological study that found an association between low birthweight and the development of cardiovascular disease in adult life. Other epidemiological studies have shown that perinatal characteristics, particularly birthweight, are associated with postnatal health [2,3].

Since about 1980, the average birthweight for both sexes in Japan has been declining. The proportion of babies with a birthweight < 2500 g has increased from 4.6 to 8.1% for singleton births and from 52.5 to 71.4% for multiple births from 1975 to 2019; this situation is unique among developed countries [4]. The main causes are believed to be inadequate nutritional intake in pregnant women and an increase in complications such as gestational hypertension owing to higher gestational age [5], and there is concern that future lifestyle-related diseases such as obesity, type 2 diabetes, and cardiovascular disease will be more prevalent in children who have been exposed to such a perinatal environment. Yet, few large-scale studies have been performed on the Japanese population regarding this.

Since 1947, pregnant women have been provided the Maternal and Child Health Handbook (MCHH), which is used by practically all mothers and children in Japan. It has been demonstrated to be useful, nationally and internationally, for improving the health of children since it records the outcomes of health checkups and provides health guidance for pregnant women, infants and young children, as well as various types of information required during this period [6]. It also helps to evaluate the presence or absence of intrauterine hypoplasia based on maternal information and physical measurements at birth.

We initially examined the relationship between current physical measurements and those at birth and infancy in 35 adults who underwent health checkups at a clinic. For this, we conducted a web-based questionnaire survey using MCHHs for participants who underwent health checkups as adults. Our analysis of their health checkup data revealed a significant association between adult body mass index (BMI) and BMI at 3 years of age as well as the difference between BMI z-scores at 3 and 1.5 years. This suggests that changes in physiques at 1.5 and 3 years of age, when infant health checkups are performed in Japan, could be a predictor of the future risk of obesity and leanness. Therefore, we planned a study at our facility to analyze a larger cohort of participants.

## 2. Materials and Methods

### 2.1. Study Population

Participants were recruited from among faculty members or medical graduate students aged ≥ 20 years at the Hongo-Ochanomizu Campus of Juntendo University in Tokyo who underwent a health checkup in June 2022. Informed consent was obtained from the study participants via the web, and at the same time, a questionnaire-based survey was conducted on the information available in the MCHH, including anthropometric measurements at birth and 1.5 and 3 years of age.

**Figure 1:**
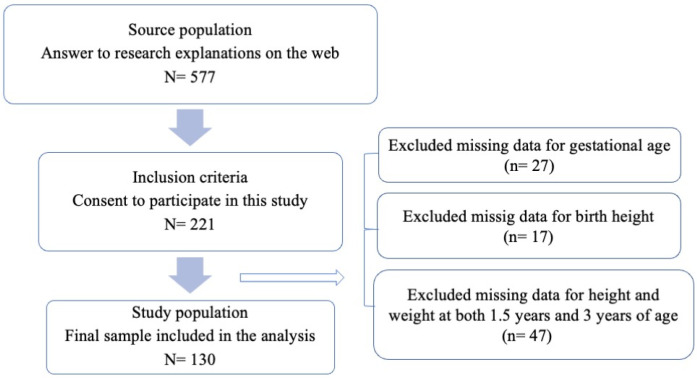
Flow chart for study population inclusion.

Of the 577 respondents, 221 consented to participate in this study. We excluded participants with missing data on gestational age (n = 27), height at birth (n = 17), and height and weight at 1.5 and 3 years of age (n = 47). The final number of participants included in the analysis was 130 (Figure 1).

### 2.2. Data Collection

Among the outcomes of the health checkups, clinical data such as anthropometric measurements, blood pressure, and blood biochemical tests related to metabolic syndromes were obtained from the database of the health administration office. Regarding the physical evaluation, BMI was calculated by dividing body weight (kg) by height (m) squared, and the BMI z-score was determined based on the standard growth chart for children from a 2000 nationwide survey [7]. The Japanese Society for Pediatric Endocrinology has developed Excel-based software for generating standard curves for both parameters (BMI and BMI z-scores), which is available on the society’s website [8]. The Ponderal index (PI), which is calculated by multiplying the birthweight (g) by 100 and then dividing the result by the cubed body length (cm), measured from the crown of the head to the heel of the foot, is an appropriate indicator of intrauterine growth restriction in the neonatal period and is also used as an index of fetal growth [9].

### 2.3. Statistical Analyses

Based on the information obtained, a simple regression analysis was performed on the association between BMI at the time of the adult health checkup and birthweight z-score; height z-score at birth; PI at birth; and BMI at birth and 1.5 and 3 years of age. In addition, the difference between BMI or BMI z-score at the ages of 1.5 and 3 years to assess the role of adiposity rebound (AR), a phenomenon represented by the second increase in BMI throughout childhood after 6 years of age, was defined as ΔBMI or ΔBMI z-score. Their relationship with BMI at the time of the adult health checkup was separately examined. We conducted multivariate analyses using multiple linear regression and calculated the regression coefficient (β) and 95% confidence intervals (CIs) considering the full model and adjusting for potential confounders such as age and sex and ΔBMI or birthweight. To investigate any factor that may influence the results, we performed a supplementary assessment by sex, based on the full model. All analyses were performed using STATA SE 16.0 (STATA Corp, College Station, TX, USA). Two-sided *P* values of less than 0.05 were considered statistically significant.

This study was approved by the Institutional Review Board at Juntendo University (project number E22-0306).

## 3. Results

The participants’ characteristics are presented in Table 1. Overall, the proportion of women was 73.1%, the median age at the health checkup was 36 (range: 22–62) years, the median BMI was 20.4 (range: 16.1–33.1) kg/m^2^, and 9.2% had a BMI of ≥25, indicative of obesity. The median gestational age at birth was 39.3 weeks; the average birthweight and height were 3135 g and 49.4 cm, respectively; the median PI was 2.6 g/cm^3^; and one case had a small gestational age. At the age of 1.5 years, the average weight and height were 10.4 kg and 79.9 cm, respectively. At the age of 3 years, the average weight and height were 14.2 kg and 95.1 cm, respectively.

The single regression analysis showed that BMI at the time of the adult health checkup was not significantly associated with birthweight z-score (β 0.26, 95% CI: −0.36–0.88), height z-score at birth (β 0.38, 95% CI: −0.19–0.96), BMI at birth (β 0.10, 95% CI: −0.45–0.65), or PI (β −0.29, 95% CI: −2.74–2.15) (Table 2).

**Figure 2:**
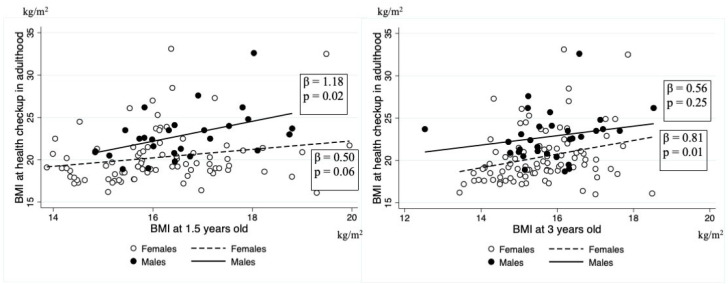
Association of anthropometric measures at 1.5 and 3 years of age with BMI in adulthood. BMI, body mass index.

**Figure 3:**
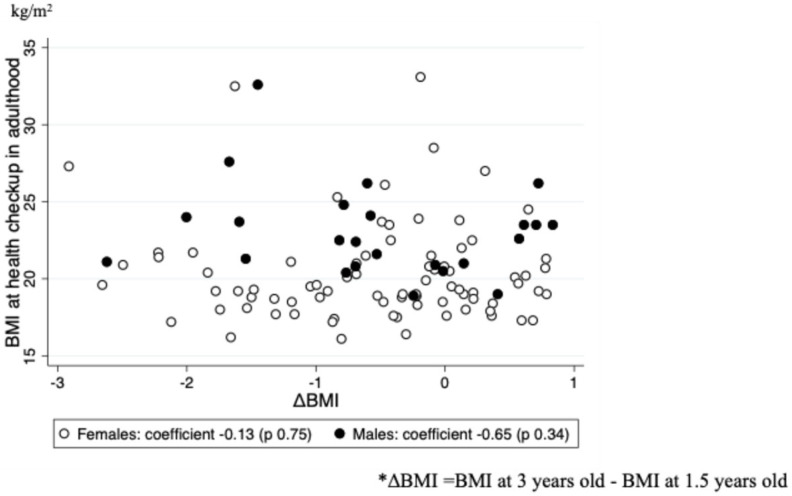
Associations between BMI in adulthood and difference in BMI from age 1.5 to 3 years. BMI, body mass index.

Figure 2 shows the results of a single regression analysis of the relationship between BMI at the time of the adult health checkup and at 1.5 and 3 years of age, respectively. Regarding the relationship between BMI in adulthood and BMI at 1.5 years of age, the regression coefficient was 0.50 (95% CI: −0.02–1.03, *p* = 0.06) for women and 1.18 (95% CI: 0.23–2.12, *p* = 0.02) for men, respectively. Regarding the relationship between BMI as an adult and BMI at 3 years of age, the regression coefficient was 0.81 (95% CI: 0.21–1.40, *p* = 0.01) for women and 0.56 (95% CI: −0.42–1.53, *p* = 0.25) for men, respectively. Furthermore, BMI at the adult health checkup was not significantly associated with ΔBMI (β −0.13, 95% CI −0.94–0.68 for women, β −0.65, 95% CI −2.03–0.74 for men) (Figure 3).

In addition, multiple regression analyses were performed to examine the relationship between BMI at the adult health checkup and anthropometric measurements at birth considering age, sex, and ΔBMI and the relationship between BMI at the adult health checkup and anthropometric measurements at ages 1.5 and 3 years considering age, sex, and birthweight. There was no significant association between BMI at the adult health checkup and anthropometric measurements at birth, but significant associations were observed between BMI z-scores at 1.5 and 3 years of age (Table 3).

When considering the relationship with each covariate related to lifestyle-related diseases other than BMI, the ratio of waist circumference to the waist-to-height ratio in adulthood was slightly but significantly associated with BMI z-score at ages 1.5 and 3 years (Table 4). Another supplemental analysis, excluding premature birth and low birthweight, yielded similar results.

## 4. Discussion

To the best of our knowledge, this is the first study in Japan to examine the relationship between BMI during adult health checkups and anthropometric measurements at birth and in infancy obtained using the MCHH. After adjusting for major confounders, a significant association was observed between BMI in adulthood and BMI z-score in early childhood.

A German cohort study based on people in their late teens reported that nearly 90% of children who were obese at 3 years of age were overweight or obese during adolescence, and among the obese adolescents, the rate of increase in BMI was greatest between the ages of two and six [10]. A British longitudinal study also reported that in young adults, BMI was more strongly associated with weight gain from 1 year and 9 months to 5 years of age [11]. In a Japanese study, it was reported that BMI at 1.5 and 3 years of age may predict the presence or absence of adiposity at 14 years of age [9]. Although infants and young children experience rapid changes in height and weight, Slining et al. [12] reported that infant BMI trajectories are associated with young adult body composition using a large birth cohort. Consistent with these observations, our study found that anthropometric measurements at 1.5 and 3 years of age are associated with those in adulthood, albeit with limited support due to the small proportion of obese individuals. This indicated the significance of identifying high-risk children during health checkups in infancy and providing appropriate guidance.

Other probable factors related to our findings include the prevalence of inadequate nutrition and lifestyle habits until the age of 3, which may affect future obesity. A systematic review reported that rapid weight gain in infancy or by 2 years of age is an important predictor of future obesity [13]. It was also reported that differences in gut bacteria between obese and non-obese adults were already observed at the age of 3 years [14]. Therefore, environmental challenges in early infancy may increase the risk of future obesity more than the anthropometric measurements at birth.

Adiposity rebound (AR) is a concept proposed by Rolland-Cachera et al. [15] in 1984 and refers to a phenomenon in which BMI rises in early infancy, stagnates or decreases around the age of 5 to 7 years, and rises again in adolescence. Early AR has been reported to be strongly associated with obesity and type 2 diabetes in adolescence and adulthood and is known to be a predictor of future obesity and metabolic syndromes [16,17]. In Japan, Ichikawa et al. [18] reported that an increase in BMI from 1.5 to 3 years is considered a useful alternative to early AR because an increase in BMI at age 3 is associated with the risk of obesity, insulin resistance, hypertension, and arteriosclerosis. However, in this study, an increase in BMI from 1.5 to 3 years of age was not an indicator of obesity in adulthood, so other factors needed to be considered. Previous reports on the association between early AR and future lifestyle disease risk have often focused on childhood and young adults [19,20,21]. Few studies have reported a direct association between early AR and obesity later during middle age after 40, as in the present study [17]. Other than AR, environmental factors, such as place of residence, physical activity, and socioeconomic background, may influence obesity at middle age [22,23,24,25].

We examined the association between BMI z-scores at 1.5 and 3 years of age and indicators related to lifestyle-related diseases such as obesity, type 2 diabetes, and cardiovascular disease in adulthood. An earlier systematic review had shown that waist-to-height ratio is a better screening tool than waist circumference and BMI for detecting risk factors for cardiometabolic disease in adults [26]. Another systematic review reported that waist-to-height ratio is a better predictor of cardiovascular disease, metabolic syndrome, and diabetes risk than BMI, waist circumference, and waist-to-hip ratio [27]. We also found in this study that the BMI z-score at 1.5 and 3 years of age was associated not only with BMI in adulthood but also with waist-to-height ratio.

A major strength of this study was that the study was conducted by collecting information from birth to adulthood about each individual using the MCHH, which is unique to Japan. The use of the MCHH is expected to have a positive effect on early childhood health because it allows healthcare providers and parents to identify problems early and implement relevant interventions, such as lifestyle modifications [28]. Ultimately, it may provide medical economic benefits by reducing the risk of obesity in the future. In addition, the comparison of anthropometric measures in adulthood and infancy was novel in our country, as it had not been explored before.

There are also some limitations that should be acknowledged. First, the study population was predominantly women and had a wide age range, and generalizability is limited owing to it being a single-institution study with relatively small sample sizes. In addition, there was a possibility that the population had a low rate of obesity at the time of the health checkup in adulthood, which might have affected the outcomes. In 2019, 33.0% of Japanese men and 22.3% of women had obesity (BMI ≥ 25 kg/m^2^) [29]. However, this study found an overall incidence of 9.2%. A unified opinion was also not obtained in this study based on sex; hence, further study with a larger sample size, including obese individuals, is warranted. Furthermore, it has been observed that in children, anthropometric measurements, as well as eating behavior, nutritional status, and social conditions, are influenced by their parents [30,31,32]; therefore, there remains a possibility that information concerning families, especially socioeconomic status, was insufficient. Furthermore, although this study used BMI for the analysis, its validity may be limited, particularly for 1.5-year-old children. Although the World Health Organization growth standards include a BMI chart from birth to 2 years of age, the Centers for Disease Control and Prevention expert panel generally agrees that many questions regarding infant BMI remain unanswered and does not recommend using BMI charts in clinical practice for children under the age of 2 [33]. Although the file used to calculate the BMI z-score is limited to Japan, it has been used as a standard guideline for many years, and there have been no reports of concern, so it is considered acceptable to use it for 1.5-year-olds. Furthermore, we believe that this study is important because it evaluated trends in BMI at two later time periods (3 years and adulthood) in addition to 1.5 years of age. Finally, the possibility of information bias owing to a lack of accuracy was suggested because it was a questionnaire-based survey. The format of the MCHH changes over time, and it differs depending on the municipality, so more reliable information could be acquired by presenting easy-to-understand explanations and examples.

## 5. Conclusions

In conclusion, we examined the relationship between anthropometric measurements in adulthood and infancy for adults who underwent a health checkup using MCHHs. BMI at the time of health checkup in adulthood was associated with BMI at 1.5 and 3 years of age. This study showed that providing appropriate guidance to children with high BMI and their parents at health checkups is important for preventing lifestyle-related diseases in adulthood.

## Figures and Tables

**Table 1:** Baseline characteristics of participants.

***N* (%)**	130 (100)
At health checkup	
Sex	
Men	35 (26.9)
Women	95 (73.1)
Age (years)	36.6 ± 9.2
BMI (kg/m^2^)	21.0 ± 3.2
≧25	12 (9.2)
<25	118 (90.8)
**At birth**	
Gestational age, weeks	39.3 ± 1.3
Preterm (<37)	1 (2.8)
Full-term (≧37)	35 (97.2)
Birthweight (g)	3135 ± 360
Birthweight z-score	0.5 ± 0.9
Birth height (cm)	49.4 ± 2.0
PI (g/cm^3^)	2.6 ± 0.2
**At 1.5 years**	
Weight (kg)	10.4 ± 1.0
Height (cm)	79.9 ± 3.6
BMI (kg/m^2^)	16.2 ± 1.3
BMI z-score	0.3 ± 0.9
**At 3 years**	
Weight (kg)	14.2 ± 1.3
Height (cm)	95.1 ± 3.5
BMI (kg/m^2^)	15.7 ± 1.0
BMI z-score	0.2 ± 0.7

Data presented as *N* (%) and mean ± standard deviation. BMI, body mass index; PI, Ponderal index.

**Table 2:** Single linear regression analysis of the associations of anthropometric measures at birth and 1.5 and 3 years of age with adult BMI.

	BMI in Adulthood		
	Coefficient	95% CI	*p*
Birthweight z-score	0.26	−0.36–0.88	0.41
Height z-score at birth	0.38	−0.19–0.96	0.19
BMI at birth	0.10	−0.45–0.65	0.72
PI at birth	−0.29	−2.74–2.15	0.81

CI: confidence interval; BMI, body mass index; PI, Ponderal index.

**Table 3:** Multivariate linear regression analysis of the associations of anthropometric measures at birth and 1.5 and 3 years of age with adult BMI.

	BMI in Adulthood		
	Coefficient	95% CI	*p*
Birthweight z-score	0.51	−0.17–1.20	0.14
Height z-score at birth	0.45	−0.15–1.05	0.14
BMI at birth	0.06	−0.58–0.71	0.84
PI at birth	−0.69	−3.49–2.11	0.63
Adjusted for age, sex, and ΔBMI			
	Coefficient	95% CI	*p*
BMI at 1.5 years old	0.53	0.04–1.02	<0.05
BMI at 3 years old	0.70	0.19–1.20	<0.01
ΔBMI	−0.12	−0.81–0.57	0.74
ΔBMI z-score	−0.05	−0.86–0.96	0.91

Adjusted for age, sex, and birthweight (kg). CI: confidence interval; BMI, body mass index; PI, Ponderal index.

**Table 4:** Multivariate linear regression results of associations between indicators related to lifestyle-related disease and BMI at 1.5 and 3 years of age.

	BMI at Age 1.5 Years			BMI at Age 3 Years		
	Coefficient	95% CI	*p*	Coefficient	95% CI	*p*
Waist circumference	1.11	−0.09–2.31	0.07	1.16	−0.10–2.41	0.07
Waist-to-height ratio	0.01	0.00–0.01	<0.05	0.01	0.00–0.02	<0.05
Systolic blood pressure	0.68	−1.22–2.59	0.48	−0.24	−2.33–1.84	0.82
Diastolic blood pressure	0.32	−1.15–1.79	0.67	0.39	−1.24–2.02	0.64
LDL cholesterol	2.24	−1.98–6.47	0.30	−0.05	−4.50–4.40	0.98
Triglyceride	1.56	−3.71–6.83	0.56	8.48	−3.27–20.23	0.16

Adjusted for age, sex and birthweight (kg). CI: confidence interval; BMI, body mass index; LDL, low-density lipoprotein.

## Data Availability

The data that support the findings of this study are available from the corresponding author [H.S.] upon reasonable request. The authors are entirely responsible for the scientific content of the paper.
